# Supplementary material for: Pyruvate Dehydrogenase Kinase 1 inhibition mediated oxidative phosphorylation enhancement in cartilage promotes osteoarthritis progression
Source: BMC Musculoskelet Disord. 2023 Jul 20;24:597. doi: 10.1186/s12891-023-06585-6 (PMC10357736; doi:10.1186/s12891-023-06585-6)
Supplement: Supplementary file 1 — Additional file 1. [file 12891_2023_6585_MOESM1_ESM.zip › BP.Gsea.1653623667859/GOBP_ACTIN_FILAMENT_BASED_TRANSPORT.html]

Details for gene set GOBP\_ACTIN\_FILAMENT\_BASED\_TRANSPORT[GSEA]

|  || Dataset | 55457\_collapsed\_to\_symbols.cls.cls #OA\_versus\_normal.cls.cls #OA\_versus\_normal\_repos |
| Phenotype | cls.cls#OA\_versus\_normal\_repos |
| Upregulated in class | normal |
| GeneSet | GOBP\_ACTIN\_FILAMENT\_BASED\_TRANSPORT |
| Enrichment Score (ES) | -0.58466595 |
| Normalized Enrichment Score (NES) | -1.7288594 |
| Nominal p-value | 0.008710802 |
| FDR q-value | 0.121757165 |
| FWER p-Value | 1.0 |
Table: GSEA Results Summary

  

Fig 1: Enrichment plot: GOBP\_ACTIN\_FILAMENT\_BASED\_TRANSPORT      
 Profile of the Running ES Score & Positions of GeneSet Members on the Rank Ordered List

  

| SYMBOL | TITLE | RANK IN GENE LIST | RANK METRIC SCORE | RUNNING ES | CORE ENRICHMENT || 1 | MYO5A | myosin VA [Source:HGNC Symbol;Acc:HGNC:7602] | 3330 | 0.218 | -0.2128 | No |
| 2 | MYO1C | myosin IC [Source:HGNC Symbol;Acc:HGNC:7597] | 3730 | 0.183 | -0.2055 | No |
| 3 | MYO1B | myosin IB [Source:HGNC Symbol;Acc:HGNC:7596] | 4425 | 0.119 | -0.2345 | No |
| 4 | MYO7A | myosin VIIA [Source:HGNC Symbol;Acc:HGNC:7606] | 4808 | 0.087 | -0.2460 | No |
| 5 | MYO1D | myosin ID [Source:HGNC Symbol;Acc:HGNC:7598] | 5091 | 0.065 | -0.2543 | No |
| 6 | MYO1F | myosin IF [Source:HGNC Symbol;Acc:HGNC:7600] | 6663 | -0.053 | -0.3651 | No |
| 7 | MYO6 | myosin VI [Source:HGNC Symbol;Acc:HGNC:7605] | 6816 | -0.064 | -0.3635 | No |
| 8 | MYO15A | myosin XVA [Source:HGNC Symbol;Acc:HGNC:7594] | 9667 | -0.289 | -0.5243 | Yes |
| 9 | FNBP1L | formin binding protein 1 like [Source:HGNC Symbol;Acc:HGNC:20851] | 10082 | -0.329 | -0.4877 | Yes |
| 10 | MYO1A | myosin IA [Source:HGNC Symbol;Acc:HGNC:7595] | 10095 | -0.330 | -0.4196 | Yes |
| 11 | SUN2 | Sad1 and UNC84 domain containing 2 [Source:HGNC Symbol;Acc:HGNC:14210] | 10366 | -0.357 | -0.3658 | Yes |
| 12 | ACTN4 | actinin alpha 4 [Source:HGNC Symbol;Acc:HGNC:166] | 10472 | -0.370 | -0.2966 | Yes |
| 13 | MYO1E | myosin IE [Source:HGNC Symbol;Acc:HGNC:7599] | 10780 | -0.404 | -0.2361 | Yes |
| 14 | SYNE2 | spectrin repeat containing nuclear envelope protein 2 [Source:HGNC Symbol;Acc:HGNC:17084] | 10841 | -0.410 | -0.1551 | Yes |
| 15 | MYO19 | myosin XIX [Source:HGNC Symbol;Acc:HGNC:26234] | 10938 | -0.423 | -0.0741 | Yes |
| 16 | MYO5C | myosin VC [Source:HGNC Symbol;Acc:HGNC:7604] | 11042 | -0.435 | 0.0089 | Yes |
| 17 | WASL | WASP like actin nucleation promoting factor [Source:HGNC Symbol;Acc:HGNC:12735] | 12207 | -0.647 | 0.0538 | Yes |
Table: GSEA details [plain text format]

  

Fig 2: GOBP\_ACTIN\_FILAMENT\_BASED\_TRANSPORT      
 Blue-Pink O' Gram in the Space of the Analyzed GeneSet

  

Fig 3: GOBP\_ACTIN\_FILAMENT\_BASED\_TRANSPORT: Random ES distribution      
 Gene set null distribution of ES for **GOBP\_ACTIN\_FILAMENT\_BASED\_TRANSPORT**
